# Palmitic Acid-Induced Hepatotoxicity in Adult Zebrafish: Molecular Mechanisms and Advances in Intervention

**DOI:** 10.3390/biology15141170

**Published:** 2026-07-16

**Authors:** Wenxuan Li, Shiwei Pan, Chi Feng, Kexin Jiang, Naer A, Jingfeng Yang

**Affiliations:** 1 College of Animal Science and Technology, Inner Mongolia Minzu University, Tongliao 028000, China; shuzhiyuyi@163.com (W.L.); psw52122@163.com (S.P.); 13634709903@163.com (K.J.); 18647953911@163.com (N.A.); 2 Department of Chemistry and Chemical Engineering, Beijing Forestry University, Beijing 100083, China; fengchi2625@163.com

**Keywords:** palmitic acid, zebrafish, fatty liver, non-alcoholic fatty liver disease

## Abstract

Palmitic acid (PA) can be used as a substitute for fish oil in aquaculture; however, excessive intake can easily cause lipotoxic liver damage in animals, and the progression of non-alcoholic fatty liver disease (NAFLD) is closely associated with it. Adult zebrafish (*Danio rerio*) share a high degree of genetic homology with humans and possess conserved lipid metabolism pathways, making them ideal model organisms for studying hepatic lipotoxicity. This paper presents a comprehensive narrative review of studies on PA-induced hepatic lipotoxicity, with a primary focus on the adult zebrafish model: it elucidates the morphological and functional abnormalities in liver tissue caused by PA exposure; explains the molecular mechanisms by which PA induces metabolic disorders and cellular stress through upregulation of lipid synthesis and inhibition of β-oxidation, which further activates inflammatory signaling pathways such as NF-κB/JNK and may ultimately contribute to liver fibrosis; and summarizes intervention strategies targeting these mechanisms. Studies using embryonic or larval zebrafish are included only as supplementary mechanistic evidence where adult data are unavailable and are clearly distinguished throughout the text. To ensure transparency, the literature search and screening process are reported in detail, although formal systematic review methodologies (e.g., PRISMA, dual independent screening, and quantitative risk-of-bias assessment) were not applied. This study aims to refine the evaluation system for PA-induced hepatic lipotoxicity, providing both theoretical support for the scientific application of PA in aquaculture and important references for elucidating the pathogenesis of human NAFLD/NASH and developing preventive and therapeutic measures.

## Simple Summary

Palmitic acid is a commonly used fish oil substitute in aquatic feed; excessive intake can cause lipotoxic liver damage in animals and is closely associated with non-alcoholic fatty liver disease. Adult zebrafish, with their high genetic homology to humans and conserved lipid metabolic pathways, serve as ideal model organisms for studying liver damage. This article provides a comprehensive narrative review of the pathological features, molecular mechanisms, and intervention strategies related to palmitic acid-induced liver injury in adult zebrafish. It elucidates how palmitic acid disrupts lipid synthesis and oxidation, induces mitochondrial and endoplasmic reticulum stress, and activates inflammatory signaling pathways, ultimately leading to liver fibrosis, and summarizes intervention strategies targeting these mechanisms. This research provides a theoretical basis for the safe application of palmitic acid in aquaculture and offers important insights for the prevention and treatment of non-alcoholic fatty liver disease in humans.

## 1. Introduction

PA, also known as hexadecanoic acid, is a saturated fatty acid (SFA) that occurs naturally in vegetable oils such as palm oil (PO, content approximately 44–52%) and other vegetable oils, as well as in animal fats such as lard (content approximately 25–30%); it is also widely distributed in human tissues and microorganisms [1]. In the aquaculture industry, due to its high yield and relatively stable supply, palm oil has gradually become one of the most commonly used lipid sources alternative to fish oil in aquafeeds over the past few decades and has been widely used in feeds for economically important aquatic animals such as carp, tilapia, shrimp, and salmon. However, diets high in PA can easily lead to excessive lipid accumulation in the livers of animals [2,3,4], causing growth retardation and hepatic steatosis in species such as red hybrid tilapia, Japanese sea bass, and red sea bream [5,6,7,8]. Its lipotoxicity has been demonstrated in various models, including hepatocytes [9], pancreatic β-cells [10], and cardiomyocytes [11], and it can cause liver tissue damage by inducing chronic inflammation, oxidative stress, and apoptosis [12,13,14].

Non-alcoholic fatty liver disease (NAFLD) ranges from benign steatosis, characterized by the accumulation of triglycerides (TG) in hepatocytes, to non-alcoholic steatohepatitis (NASH), which can progress to fibrosis, cirrhosis, and hepatocellular carcinoma. In zebrafish models, the term “NASH-like” is used throughout this review, as the full histopathological criteria for human NASH have not been uniformly established in this model. PA is the primary SFA in human plasma [15] and is widely used to construct in vitro models of hepatic lipid accumulation, providing an ideal tool for elucidating the molecular mechanisms underlying lipid overload-induced hepatotoxicity. Research findings in this area hold critical theoretical value for elucidating the pathogenesis of NAFLD.

As a model organism, the zebrafish (*Danio rerio*) shares up to 87% homology with the human genome and exhibits lipid metabolism patterns similar to those of humans. These advantages make the zebrafish an excellent model for studying lipid metabolism-related diseases, and it has been widely used in research fields such as toxicology, developmental biology, and nutrition. This review is deliberately restricted to adult zebrafish based on the following considerations: the adult liver possesses fully developed hepatic lobules, mature hepatocyte polarity, and complete metabolic enzyme systems, which are essential for recapitulating the complex pathophysiology of NAFLD, including steatosis, inflammation, and fibrosis [16]; PA-induced hepatic lipotoxicity in adult zebrafish is typically achieved through dietary intervention, a route that closely models human oral exposure and chronic metabolic stress, whereas embryonic/larval models rely on waterborne absorption that bypasses gastrointestinal digestion and enterohepatic circulation, thereby limiting their translational relevance to nutritional and metabolic research [17]; adult zebrafish also support long-term exposure paradigms and repeated sampling (blood, liver tissue) for longitudinal assessment of disease progression and intervention efficacy—experimental designs that are not feasible in larvae [17]. Accordingly, embryonic or larval zebrafish studies are cited in this review only as supplementary evidence and are clearly distinguished in the text.

Studies have shown that PA induces pathological phenomena in adult zebrafish, including increased liver volume and weight, steatosis, hyperlipidemia, hyperglycemia, and liver damage [17,18]. The underlying molecular mechanism primarily involves upregulating fatty acid synthase (FAS) gene expression and inhibiting carnitine palmitoyltransferase 1 (CPT1), thereby increasing lipid synthesis in hepatocytes. This further impedes β-oxidation, leading to hepatic lipid degeneration and associated inflammatory responses, ultimately resulting in hepatocyte apoptosis.

To date, systematic studies and summaries of the lipotoxic effects of dietary PA, particularly its role in inducing liver damage, remain scarce in fish [3]. Using zebrafish as a model organism to investigate the liver-targeted effects of PA not only aids in deeply elucidating its metabolic regulatory mechanisms in aquatic organisms and clarifying the substance’s impact on fish growth, development, and overall health—thereby providing theoretical support for the scientific application of PA in aquaculture—but also offers important insights for research into the pathogenesis of human metabolic diseases such as NAFLD. In view of the above, this review systematically summarizes research findings on PA-induced hepatotoxicity in zebrafish, covering its pathological manifestations, molecular regulatory mechanisms, and potential intervention strategies. The aim is to establish a comprehensive evaluation system for PA hepatotoxicity to promote the coordinated development of the aquaculture industry, ecotoxicology, and research on metabolic diseases.

## 2. Methods

We performed a literature search in Web of Science, PubMed, and Scopus from database inception to 30 June 2026, to identify relevant studies on palmitic acid (PA)-induced hepatic lipotoxicity in adult zebrafish. The search strategy employed a combination of controlled vocabulary and free-text terms, with core keywords encompassing research subjects, interventions, and pathological mechanisms and outcomes.

### 2.1. Search Strings

#### 2.1.1. Web of Science (Field: Topic, TS)

TS = (zebrafish OR adult zebrafish)

AND TS = (“palmitic acid” OR hexadecanoic acid OR “saturated fatty acid”)

AND TS = (“liver injury” OR hepatotoxicity OR “hepatic lipotoxicity” OR steatosis OR “fatty liver” OR NAFLD OR NASH OR MASLD OR fibrosis OR cirrhosis OR “extracellular matrix deposition” OR “hepatic stellate cell”)

AND TS = (“lipid metabolism” OR “metabolic disorder” OR “β-oxidation” OR “fatty acid oxidation” OR “mitochondrial dysfunction” OR “oxidative stress” OR “reactive oxygen species” OR “cellular stress” OR “ER stress” OR “endoplasmic reticulum stress” OR “unfolded protein response” OR autophagy OR lysosome OR p62 OR “signaling pathway” OR “inflammatory response” OR “inflammatory cytokine” OR “inflammatory signaling” OR epigenetics OR methylation OR microRNA OR “long non-coding RNA” OR sirtuin OR “metabolic memory”)

AND TS = (“lipid synthesis inhibitor” OR antioxidant OR “traditional Chinese medicine extract” OR “plant extract” OR “natural compound” OR “intervention strategy” OR “single-cell sequencing” OR transcriptomics OR metabolomics OR “multi-omics” OR “gut-liver axis” OR “high-fat diet”)

#### 2.1.2. PubMed (Field: Title/Abstract)

((zebrafish [Title/Abstract] OR adult zebrafish [Title/Abstract])

AND (“palmitic acid” [Title/Abstract] OR hexadecanoic acid [Title/Abstract] OR “saturated fatty acid” [Title/Abstract])

AND (“liver injury” [Title/Abstract] OR hepatotoxicity [Title/Abstract] OR “hepatic lipotoxicity” [Title/Abstract] OR steatosis [Title/Abstract] OR “fatty liver” [Title/Abstract] OR NAFLD [Title/Abstract] OR NASH [Title/Abstract] OR MASLD [Title/Abstract] OR fibrosis [Title/Abstract] OR cirrhosis [Title/Abstract] OR “hepatic stellate cell” [Title/Abstract])

AND (“lipid metabolism” [Title/Abstract] OR “metabolic disorder” [Title/Abstract] OR “β-oxidation” [Title/Abstract] OR “fatty acid oxidation” [Title/Abstract] OR “mitochondrial dysfunction” [Title/Abstract] OR “oxidative stress” [Title/Abstract] OR “reactive oxygen species” [Title/Abstract] OR “cellular stress” [Title/Abstract] OR “ER stress” [Title/Abstract] OR “endoplasmic reticulum stress” [Title/Abstract] OR “unfolded protein response” [Title/Abstract] OR autophagy [Title/Abstract] OR lysosome [Title/Abstract] OR p62 [Title/Abstract] OR “inflammatory response” [Title/Abstract] OR “inflammatory cytokine” [Title/Abstract] OR “inflammatory signaling” [Title/Abstract] OR epigenetics [Title/Abstract] OR methylation [Title/Abstract] OR microRNA [Title/Abstract] OR “long non-coding RNA” [Title/Abstract] OR sirtuin [Title/Abstract] OR “metabolic memory” [Title/Abstract])

AND (antioxidant [Title/Abstract] OR “natural compound” [Title/Abstract] OR “plant extract” [Title/Abstract] OR “traditional Chinese medicine extract” [Title/Abstract] OR “intervention strategy” [Title/Abstract] OR “gut-liver axis” [Title/Abstract] OR “high-fat diet” [Title/Abstract] OR transcriptomics [Title/Abstract] OR metabolomics [Title/Abstract] OR “multi-omics” [Title/Abstract]))

#### 2.1.3. Scopus (Field: TITLE-ABS-KEY)

TITLE-ABS-KEY (zebrafish OR adult zebrafish)

AND TITLE-ABS-KEY (“palmitic acid” OR hexadecanoic acid OR “saturated fatty acid”)

AND TITLE-ABS-KEY (“liver injury” OR hepatotoxicity OR “hepatic lipotoxicity” OR steatosis OR “fatty liver” OR NAFLD OR NASH OR MASLD OR fibrosis OR cirrhosis OR “extracellular matrix deposition” OR “hepatic stellate cell”)

AND TITLE-ABS-KEY (“lipid metabolism” OR “metabolic disorder” OR “β-oxidation” OR “fatty acid oxidation” OR “mitochondrial dysfunction” OR “oxidative stress” OR “reactive oxygen species” OR “cellular stress” OR “ER stress” OR “endoplasmic reticulum stress” OR “unfolded protein response” OR autophagy OR lysosome OR p62 OR “inflammatory response” OR “inflammatory cytokine” OR “inflammatory signaling” OR epigenetics OR methylation OR microRNA OR “long non-coding RNA” OR sirtuin OR “metabolic memory”)

AND TITLE-ABS-KEY (“lipid synthesis inhibitor” OR antioxidant OR “traditional Chinese medicine extract” OR “plant extract” OR “natural compound” OR “intervention strategy” OR transcriptomics OR metabolomics OR “multi-omics” OR “gut-liver axis” OR microbiota OR “high-fat diet”)

### 2.2. Supplementary Search Strategy

Given the high conservation of PA-induced lipotoxic signaling networks (e.g., SREBP-1c/PPAR-α/NF-κB, epigenetic modifications, autophagy, and ER stress) between zebrafish and mammals, we supplemented the main searches by (1) manually screening reference lists of retrieved zebrafish studies for key mechanistic or highly cited studies regardless of model organism; (2) conducting targeted topic searches without the “zebrafish” filter for specific mechanisms (e.g., epigenetic regulation, AMPK/mTOR) or interventions (e.g., curcumin, resveratrol, silymarin); and (3) mining high-impact reviews for further relevant primary studies.

### 2.3. Literature Screening Process

**Figure 1:**
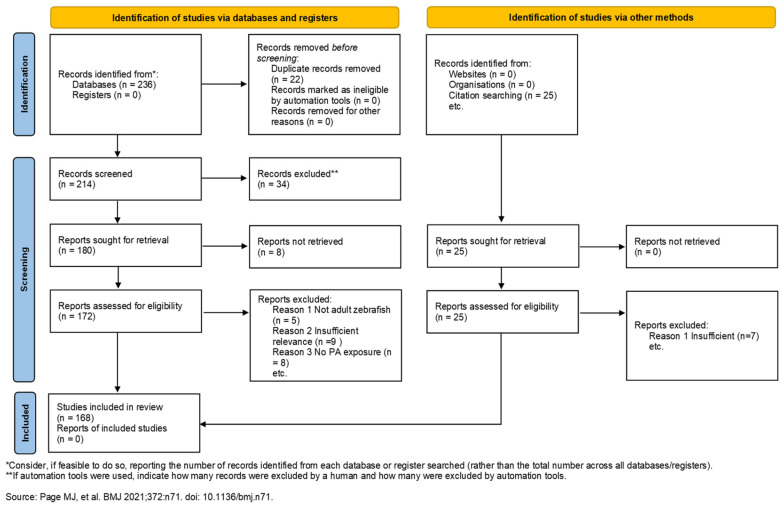
PRISMA 2020 flow diagram of the study selection process. Footnotes: The diagram shows the number of records identified, screened, assessed for eligibility, and included in the final review. Reasons for exclusion at each stage are provided in the relevant boxes. Adapted from Page et al., 2021 [19].

One reviewer (W.L.) performed the initial screening of titles, abstracts, and full texts against the eligibility criteria. To minimize selection bias, all candidate articles and excluded articles were subsequently cross-verified by three independent collaborators (K.J., N.A., and S.P.), and any disagreements regarding inclusion were discussed and resolved by consensus with the senior author (J.Y.). While this process involved multiple reviewers, we acknowledge that formal independent dual screening with blinded reviewers was not implemented, which constitutes a limitation of this review (see Section 6.1 Limitations). The screening process is summarized in the PRISMA 2020 flow diagram (Figure 1), and reasons for exclusion at the full-text stage are listed in the diagram.

The initial database search yielded 236 records (Web of Science: 72, PubMed: 101, Scopus: 63). After removal of duplicates, 22 records were excluded, leaving 214 articles. Following title and abstract screening, 34 articles were excluded as irrelevant to the topic, leaving 180 articles for full-text retrieval. Of these, 8 articles could not be obtained, and the remaining 172 articles underwent full-text review. Based on the eligibility criteria, 22 articles were excluded, leaving 150 articles.

Supplementary searches (manual reference list screening, targeted topic searches, and mining of high-impact reviews) identified an additional 25 records. Based on the eligibility criteria, 7 articles were excluded, leaving 18 articles.

Ultimately, the 150 articles from database sources and the 18 articles from supplementary sources were combined, resulting in a total of 168 articles included in this review.

### 2.4. Inclusion and Exclusion Criteria

Inclusion criteria: (1) studies using zebrafish as the experimental model for palmitic acid exposure or lipotoxicity-related experiments; (2) studies reporting liver injury, lipid accumulation, and related molecular mechanisms; (3) for conserved molecular mechanisms or intervention targets that have been validated in zebrafish, seminal studies from mammalian models (mice, rats) and cell lines were also included as supplementary mechanistic references; (4) original research and review articles published in English and peer-reviewed.

Exclusion criteria: studies unrelated to hepatic response, involving other species or fatty acids, or lacking full-text access.

### 2.5. Evidence Hierarchy and Source Attribution

In this review, findings derived from different experimental models are presented with explicit source attribution. Studies directly using adult zebrafish are considered the primary evidence for PA-induced hepatotoxicity in this model and form the core of our mechanistic and interventional synthesis. Evidence from zebrafish larvae/embryos, other fish species, mammalian models (mice, rats), and in vitro cell systems is cited for the following purposes: (1) to illustrate conserved molecular pathways that have been validated in adult zebrafish; (2) to generate hypotheses for mechanisms not yet fully characterized in adult zebrafish; and (3) to provide background or comparative context. In the main text, we have used explicit model-specifying terms (e.g., “in mouse hepatocytes”, “in rat models”, “in zebrafish larvae”) to clearly distinguish the source of each piece of evidence, ensuring that statements presented as direct evidence for adult zebrafish are supported exclusively by adult zebrafish studies. When a mechanism or intervention has not been directly demonstrated in adult zebrafish but is supported by other models, this is explicitly noted in the text.

## 3. Pathological Changes in the Livers of Adult Zebrafish Induced by PA

As PA is a common alternative lipid source in aquatic feed, its excessive accumulation in the livers of adult zebrafish disrupts the liver’s normal physiological balance and triggers a series of pathological changes. These lesions show a progressive worsening trend as PA exposure dose increases and exposure duration lengthens, as evidenced by three specific dimensions: tissue morphology, cellular structure, and functional indicators.

**Figure 2:**
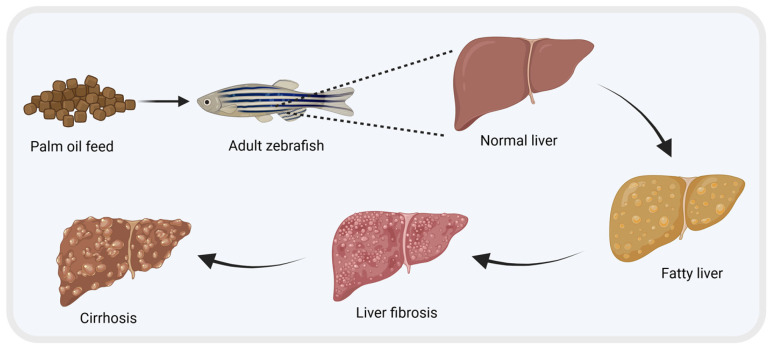
Pathological changes in zebrafish liver damage induced by PA. Footnotes: Progressive pathological changes in adult zebrafish liver after PA exposure, from tissue to cellular to functional levels. Arrows indicate progression. Data summarized from studies on PA-induced hepatic steatosis and injury in adult zebrafish [17,20]. Created in BioRender. Li, W. (2026). https://biorender.com/5gv9pgm (accessed on 21 April 2026).

The process of PA-induced liver injury in zebrafish is shown in Figure 2:

### 3.1. Histological Damage to the Liver

Numerous studies have demonstrated that dietary exposure to PA leads to significant morphological changes in the livers of adult zebrafish, exhibiting dose-dependent characteristics. Zebrafish fed high levels of PA exhibit weight gain, hyperlipidemia, hyperglycemia, steatosis, and a phenotype consistent with liver damage [17]. Pathological examination reveals organ adhesions, a yellowish-brown discoloration of the liver, a surface layer of fat, and liver tissue that is fragile and less elastic. These findings are associated with cellular swelling due to massive lipid accumulation in hepatocytes and compensatory tissue hyperplasia.

PA-treated zebrafish livers exhibited typical features of steatosis, with lipid vacuoles of varying sizes appearing within hepatocytes [20]; the cell nuclei were compressed into flattened or irregular shapes, and the normal morphological structure of the hepatocytes was severely disrupted. Existing studies have demonstrated that a diet high in saturated fatty acids induces extensive lipid accumulation in hepatocytes of adult zebrafish [17,20,21]. Exposure to high concentrations of PA also induces inflammatory infiltration in liver tissue. In H&E-stained sections, inflammatory cells—primarily macrophages—are observed aggregating in the spaces between hepatic sinusoids and around necrotic hepatocytes, forming localized inflammatory foci [22]; it also leads to hepatocyte necrosis in zebrafish, manifested by increased cytoplasmic eosinophilia, nuclear condensation or dissolution, and the appearance of red blood cells in the interstitial spaces [17]; insoluble protein inclusions have been shown to play a significant role in lipotoxic liver injury induced by saturated fatty acids such as PA [20]. Currently, multiple studies have identified this marker as a key characteristic of lipotoxic liver injury and a potential therapeutic target [23,24].

### 3.2. Structural Damage to Liver Cells

PA can cause multi-target damage to structures such as hepatocyte mitochondria and the endoplasmic reticulum. As the central organelles of energy metabolism in hepatocytes, mitochondria are the primary target of PA’s lipotoxic effects [25], and the resulting damage involves pathological mechanisms such as oxidative stress, impaired ATP synthesis, increased ROS production, and apoptosis [26]. Mitochondria in PA-treated hepatocytes exhibit swelling, with some taking on a spherical shape; the outer membrane shows localized rupture or dissolution, and the cristae structure of the inner membrane is disorganized and fragmented. In severe cases, the cristae disappear completely, leaving only vacuolar membrane structures, and granules with uneven electron density appear within the matrix.

The endoplasmic reticulum (ER) is an organelle in hepatocytes responsible for protein folding, lipid synthesis, and the regulation of calcium homeostasis; ER stress is a key factor driving the onset and progression of NASH [27]. Following PA exposure, misfolded or unfolded proteins accumulate within the endoplasmic reticulum lumen, forming clumps of high electron density. Concurrently, the number of endoplasmic reticulum-mitochondria contact sites (MAMs) increases, leading to massive Ca^2+^ release from the endoplasmic reticulum, which induces ER stress. This Ca^2+^ then transfers to the mitochondria, exacerbating mitochondrial damage and creating a vicious cycle of ER stress and mitochondrial damage [28].

Changes in the morphology and distribution of lipid droplets are a direct manifestation of PA-induced hepatic steatosis [29]. Following PA treatment, the number of lipid droplets in zebrafish hepatocytes increases, and their volume expands, compressing surrounding organelles and disrupting their arrangement; if the lipid droplet membrane structure is compromised, lipid leakage occurs, further damaging the surrounding organelles. Lipidomic analyses have shown that aggregated lipid droplets contain higher levels of saturated fatty acids and lower levels of unsaturated fatty acids [30]; decreased expression of proteins associated with the lipid droplet surface leads to enhanced mitochondrial oxidative capacity in hepatocytes, resulting in lipid droplet fusion and rupture, which exacerbates lipotoxic damage to hepatocytes [31].

### 3.3. Abnormal Liver Function Test Results

PA-induced pathological damage to the livers of adult zebrafish directly leads to functional disorders, resulting in abnormal changes in serum and liver tissue markers related to liver function, lipid metabolism, and oxidative stress.

Liver enzyme levels are sensitive indicators of hepatocyte damage. PA causes a significant increase in serum alanine transaminase (ALT), aspartate transaminase (AST), and alkaline phosphatase (ALP) levels [32,33,34], indicating increased permeability of the hepatocyte membrane and the release of intracellular enzymes into the bloodstream, suggesting hepatocyte degeneration or necrosis. In addition, PA exposure also leads to elevated serum gamma-glutamyl transferase (GGT) activity, suggesting damage to the hepatobiliary system and impaired bile excretion, indicating that the injury has extended to the biliary tract.

Excessive accumulation of PA in the liver disrupts the balance of lipid metabolism in zebrafish livers, leading to abnormal metabolic parameters. First, the levels of triglycerides (TG) and total cholesterol (TC) in liver tissue significantly increase, indicating accelerated lipid accumulation within hepatocytes [20]; simultaneously, serum lipid levels also become abnormal, suggesting that PA may induce systemic lipid metabolism disorders and increase the risk of cardiovascular disease [35].

PA exposure can also induce oxidative stress in the zebrafish liver, leading to a significant increase in reactive oxygen species (ROS) production [36], which impairs the function of the antioxidant enzyme system. This results in reduced activity of superoxide dismutase (SOD), catalase (CAT), and glutathione peroxidase (GPX), causing an imbalance between oxidative and antioxidant functions [37,38]; concurrently, elevated levels of the lipid peroxidation product malondialdehyde (MDA) further exacerbate hepatocyte damage, a finding confirmed in studies using high-fat diet models rich in saturated fatty acids such as PA [39].

In addition, PA exposure triggers an inflammatory response in the liver; PA and its ester derivatives can upregulate the mRNA and protein expression levels of pro-inflammatory factors such as tumor necrosis factor-α (TNF-α), interleukin-1β (IL-1β), and interleukin-6 (IL-6) [40,41], while simultaneously promoting increased phosphorylation of proteins associated with inflammatory signaling pathways (such as NF-κB p65 and p-JNK), indicating that PA promotes the release of inflammatory factors by activating inflammatory signaling pathways, exacerbates hepatic inflammatory damage, and could drive the progression of liver lesions from steatosis to steatohepatitis.

## 4. The Molecular Mechanism by Which PA Causes Liver Damage

PA primarily mediates the onset and progression of lipotoxic liver injury in zebrafish through mechanisms such as mitochondrial dysfunction, endoplasmic reticulum stress, oxidative stress, inflammatory responses, and apoptosis [42]. Lipid metabolism disorders mark the starting point of this injury process; increased synthesis of fatty acids and triglycerides in the liver, coupled with impaired efflux, leads to excessive lipid accumulation [43,44]. Excess lipids exacerbate the metabolic burden on mitochondrial and peroxisomal β-oxidation, triggering uncontrolled lipid peroxidation. Consequently, a large amount of ROS is generated within hepatocytes, antioxidant substances are continuously depleted, and cytotoxic substances accumulate, further inducing the massive release of pro-inflammatory cytokines [45,46,47]. Simultaneously, liver injury also activates resident liver macrophages (Kupffer cells, KCs). The combined effects of these pathological injuries ultimately lead to NASH.

### 4.1. Metabolic Disorders: Altered Expression of Genes Related to Lipid Metabolism in the Liver

As a saturated fatty acid, PA can disrupt the balance between lipid synthesis and breakdown by regulating the expression of key genes involved in hepatic lipid metabolism, thereby triggering pathological changes such as hepatic steatosis.

#### 4.1.1. Upregulation of Lipid Synthesis

Sterol Regulatory Element-Binding Protein-1c (SREBP-1c) is a key transcription factor that upregulates lipid synthesis in hepatocytes. Localized in the endoplasmic reticulum, it senses intracellular cholesterol and fatty acid levels [48]. When PA enters hepatocytes, it first triggers endoplasmic reticulum stress, leading to a decrease in membrane fluidity; simultaneously, the binding of palmitoyl-CoA (PA-CoA) to Insig-1 is weakened, lifting the inhibition on lipid synthesis and promoting the cleavage of SREBP-1c by S1P/S2P proteases into its active nuclear form (nSREBP-1c), which then enters the cell nucleus.

Once inside the cell nucleus, nSREBP-1c binds to the sterol regulatory element (SRE), directly activating the lipid-synthesis-related genes encoding fatty acid synthase (FAS), acetyl-CoA carboxylase 1 (ACC1), and stearoyl-CoA desaturase 1 (SCD1) [49,50]. FAS is a key enzyme in de novo fatty acid synthesis, catalyzing the conversion of acetyl-CoA to PA; its upregulation directly increases the rate of fatty acid synthesis in the liver [51]. ACC1, the rate-limiting enzyme in the pathway, catalyzes the conversion of acetyl-CoA to malonyl-CoA, providing the substrate for FAS; SCD1 introduces a double bond into PA, converting it to palmitoleic acid, thereby facilitating the synthesis and transport of triglycerides (TG) [52]. Furthermore, mTORC1 and PPAR-γ can synergistically enhance the activity of SREBP-1c [53,54]: mTORC1 is reported to phosphorylate Lipin-1 to prevent its nuclear translocation and inhibit SREBP-1c, while also directly phosphorylating SREBP-1c to promote its maturation; although PPAR-γ expression in hepatocytes is lower than in adipocytes, it can still form heterodimers with RXR to enhance SREBP-1c promoter activity and amplify regulatory signals [55].

PA induces endoplasmic reticulum stress and upregulates insulin signaling, activating mTORC1 and PPAR-γ, which in turn promote nSREBP-1c-mediated transcription of FAS, ACC1 and SCD1, forming a complete metabolic pathway: ACC1 catalyzes the conversion of acetyl-CoA to malonyl-CoA, which is then synthesized into PA by FAS and converted into palmitoleic acid by SCD1, ultimately contributing to TG synthesis [56,57,58]. If this pathway is overactivated, accumulated succinyl-CoA competitively inhibits CPT1 and blocks β-oxidation at the enzyme activity level; simultaneously, an increased proportion of saturated lipids in the endoplasmic reticulum membrane exacerbates ER stress, while mechanical compression of lipid droplets leads to nuclear displacement and mitochondrial damage, which may further exacerbate ER stress and cellular damage, potentially contributing to inflammation and cell death in the current experimental model.

#### 4.1.2. Downregulation of β-Oxidation and Mitochondrial Dysfunction

β-oxidation is the central pathway of fatty acid catabolism; its downregulation leads to lipid accumulation and can cause cellular dysfunction or even cell death via apoptosis and necrosis [59]. The core mechanism involves malonyl-CoA accumulation and the dual inhibition of carnitine palmitoyltransferase 1A (CPT1A) and acyl-CoA oxidase 1 (ACOX1).

CPT1 is the rate-limiting enzyme in fatty acid β-oxidation [60], catalyzing the conversion of long-chain acyl-CoA to acyl-carnitine for transport into mitochondria, where the acyl group undergoes β-oxidation to generate energy. Downregulation of CPT1 expression directly inhibits β-oxidation and reduces the flux of fatty acid oxidation [61]. Moreover, CPT1A deficiency can induce energy metabolism disorders associated with myocardial lipotoxicity [62,63]. Malonyl-CoA acts as an endogenous inhibitor of CPT1 by competitively binding to it and suppressing its enzymatic activity at the post-translational level. Importantly, this competitive inhibition does not reduce CPT1 protein expression; rather, accumulation of malonyl-CoA primarily reinforces FAS-mediated fatty acid synthesis, forming a vicious cycle that exacerbates lipotoxicity.

ACOX1 is the rate-limiting enzyme for the oxidation of very long-chain fatty acids (VLCFAs) in peroxisomes; its downregulation blocks this pathway, leading to VLCFA accumulation [64]. Studies have shown that upregulation of miR-103-3p in the livers of NASH patients, high-fat diet-fed mice, and PA-treated hepatocytes suppresses ACOX1 expression, thereby exacerbating hepatic lipid accumulation [65]. In addition to this microRNA-mediated regulation, the nuclear receptor PPAR-α is a key transcriptional regulator of CPT1 and ACOX1 expressions. It binds to specific DNA sequences, PPREs, to regulate the expression of target genes including CPT1A, ACOX1, PGC-1α, and CYP4A [66]. When PA disrupts lipid metabolism in zebrafish livers, leading to lipid accumulation and inflammation, PPAR-α gene expression decreases. This subsequently downregulates CPT1 and ACOX1 at the transcriptional level, exacerbating PA-induced hepatic lipotoxicity. Downregulation of CYP4A suggests impaired peroxisomal function, while downregulation of PGC-1α leads to reduced mitochondrial biogenesis, decreased membrane potential, and increased ROS production [67].

PA can also directly induce mitochondrial dysfunction, manifested as a significant decrease in mitochondrial membrane potential (MMP) [68]. The deacetylase Sirtuin 3 (SIRT3) is involved in regulating mitochondrial energy metabolism [69], and its deficiency leads to reduced ATP levels and elevated ROS levels [70]. In one study, SIRT3 activity was significantly reduced in liver cells from rats exposed to PA, accompanied by elevated ROS levels and reduced mitochondrial autophagy-mediated clearance, indicating that PA-induced downregulation of SIRT3 is a key contributor to the loss of mitochondrial membrane potential and apoptosis [71].

The dual inhibition of mitochondrial and peroxisomal β-oxidation, coupled with increased fatty acid synthesis driven by SREBP-1c, leads to TG accumulation and insufficient fatty acid oxidation, further exacerbating lipid metabolism disorders. This process is prevalent in metabolic diseases such as obesity, NASH, and diabetes; therefore, targeting the ACC1/PPAR-α/CPT1A axis may serve as a potential intervention strategy. Downregulation of mitochondrial β-oxidation can also shift myocardial energy metabolism from fatty acids to glucose; however, glucose oxidation is highly inefficient, readily leading to ATP deficiency and oxidative stress, thereby increasing the risk of heart failure [72].

#### 4.1.3. PA-Induced Metabolic Imbalance in the Liver Leads to the Formation of “Metabolic Memory” and Epigenetic Changes

Consistent with this epigenetic lipid-disrupting phenotype, a zebrafish toxicology study confirmed that HFPO-TA exposure rewires hepatic epigenomes to trigger persistent hepatic lipid dysregulation [73]. Following a short-term metabolic imbalance in the liver induced by PA, the inhibited state of β-oxidation can be sustained for an extended period even as subsequent stimulation gradually diminishes, primarily through two key mechanisms: DNA methylation and microRNA regulation.

On the one hand, PA is suggested to induce high expression of DNA methyltransferase 1 (DNMT1). DNMT1 has been reported to catalyze the transfer of methyl groups to the promoter region of the PPAR-α gene, forming a methylation barrier that inhibits PPAR-α transcription and silences the gene, thereby continuously suppressing the β-oxidation process [73]. Since DNMT1 possesses the ability to recognize “hemi-methylated DNA”, it maintains the methylation status of the PPAR-α promoter during cell division; therefore, even if PA exposure decreases, the inhibition of β-oxidation is still transmitted to daughter cells. On the other hand, PA has been shown to upregulate the expression of the pro-apoptotic non-coding microRNA (miRNA) miR-34a by activating the p53 or NF-κB pathways as reported in mammalian cancer cell models [74]. It may directly bind to the mRNA of the deacetylase SIRT1, thereby inhibiting SIRT1 expression in mammalian cell lines [75,76]. Downregulation of SIRT1 leads to increased acetylation levels and reduced activity of PGC-1α, ultimately resulting in sustained inhibition of β-oxidation [77]. DNMT1-mediated DNA methylation blocks the transcription of genes involved in β-oxidation, while the miR-34a/SIRT1 pathway inhibits the activation of mitochondrial function; the interaction between these two mechanisms ultimately leads to a complete collapse of fatty acid oxidation capacity. A study identified 8 differentially expressed lncRNAs with human homologs in a high-cholesterol-diet NAFLD zebrafish model. These lncRNAs may be involved in the development of NAFLD via epigenetic mechanisms, including chromatin remodeling and transcriptional regulation mediated by lncRNAs [78].

### 4.2. Cellular Stress: The Triple Threat of Oxidative Stress, Endoplasmic Reticulum Stress, and Autophagy

In PA-induced lipotoxicity in zebrafish hepatocytes, cellular stress represents the core response of hepatocytes to lipid overload. This primarily includes oxidative stress, endoplasmic reticulum stress, and mitochondrial stress, which interact to further disrupt cellular homeostasis, thereby creating conditions conducive to subsequent inflammation and apoptosis.

#### 4.2.1. Oxidative Stress

The essence of oxidative stress lies in increased ROS production and reduced antioxidant capacity, leading to the accumulation of ROS within cells and their attack on cellular components; excessive lipid accumulation exacerbates this process, thereby inducing cell apoptosis [79]. ROS primarily include superoxide anions (O_2_^−^), hydrogen peroxide (H_2_O_2_), and hydroxyl radicals (·OH). Under normal physiological conditions, small amounts of these species can participate in signal transduction, but excessive levels become “cytotoxic”. Studies have shown that as PA concentration increases and treatment duration extends, ROS production significantly increases [80]. PA primarily disrupts the ROS balance through two mechanisms: by actively activating ROS-producing pathways and by passively inhibiting the antioxidant system.

NADPH oxidase (NOX) plays a key role in activating the ROS production pathway. Under normal conditions, NOX is primarily activated in immune cells such as neutrophils to kill bacteria; however, PA can abnormally activate the NOX1/2/5 subtypes in hepatocytes, catalyzing the conversion of O_2_ into O_2_^−^, which is then converted into the more toxic H_2_O_2_ or ·OH, thereby amplifying oxidative stress. This is the primary source of PA-induced ROS production in the liver. In addition, xanthine oxidoreductase (XOR) may also be involved as suggested by mammalian studies. Under normal physiological conditions, XOR is present as xanthine dehydrogenase (XDH), which efficiently catalyzes purine metabolism without producing ROS, thereby releasing only energy [81]. PA has been reported to induce the conversion of XDH to xanthine oxidase (XO) via oxidative stress signaling, causing XO to directly transfer electrons to O_2_ during the catalytic conversion of xanthine to uric acid, thereby generating O_2_^−^ and H_2_O_2_. This transforms the normal metabolic pathway into a ROS-producing one, as demonstrated in mammalian systems, further exacerbating the accumulation of reactive oxygen species [82].

In terms of inhibiting the antioxidant system, PA can reduce the activity of the three major antioxidant enzymes in cells. The cellular antioxidant defense system converts highly toxic O_2_^−^ into less toxic H_2_O_2_ via SOD; CAT breaks down H_2_O_2_ into H_2_O and O_2_; and finally, glutathione peroxidase (GSH-Px) clears H_2_O_2_ and lipid peroxides. PA simultaneously inhibits the activity of these three enzymes, preventing ROS from being effectively cleared.

When excessive ROS are generated, they attack lipids, proteins, and DNA, producing new toxic substances. For example, ROS attack unsaturated fatty acids in cell membranes, triggering lipid peroxidation and generating substances such as MDA and 4-hydroxy-2-nonenal (4-HNE) [83]; simultaneously, ROS oxidize proteins, producing protein carbonyl products. These products further impair DNA and protein function, inducing the production of more ROS and creating a vicious cycle. This process is the key mechanism by which oxidative stress transitions from acute injury to chronic pathology, and it is also one of the core pathological foundations underlying the progression of PA-induced liver injury from simple steatosis to NASH-like.

#### 4.2.2. Endoplasmic Reticulum Stress—Disordered Protein Folding

The endoplasmic reticulum (ER) serves as the site for protein synthesis, folding, and modification, and is also the primary intracellular calcium reservoir. In the liver, the ER is also involved in the assembly of very-low-density lipoproteins (VLDL) and lipid synthesis. When zebrafish liver cells are exposed to stimuli such as excessive lipid accumulation or oxidative damage, endoplasmic reticulum homeostasis is disrupted, leading to the accumulation of misfolded or unfolded proteins and disturbances in calcium ion balance-a condition known as endoplasmic reticulum stress (ER stress) [27]. This is one of the key molecular mechanisms underlying PA-induced liver injury in zebrafish. In response to ER stress, cells activate the unfolded protein response (UPR) to restore homeostasis [84]. The UPR is mediated by three transmembrane proteins-PERK, IRE1, and ATF6-which activate distinct signaling pathways to regulate protein synthesis, folding, and degradation [85]. If the stress persists or becomes too intense, the UPR cannot restore homeostasis, leading to inflammation and apoptosis, which further exacerbate liver damage.

Protein kinase R-like endoplasmic reticulum kinase (PERK) is activated when misfolded proteins accumulate; it inhibits protein synthesis by phosphorylating eukaryotic translation initiation factor 2α (eIF2α), thereby alleviating the burden on the endoplasmic reticulum. However, this simultaneously activates the transcription factor ATF4, which in turn induces the expression of the pro-apoptotic transcription factor CHOP, promoting apoptosis in hepatocytes. Studies have shown that in a PA-induced zebrafish liver injury model, excessive PERK activation exacerbates apoptosis triggered by excessive lipid accumulation [86], synergistically elevating ALT and AST levels with the caspase family and increasing the number of apoptotic bodies.

Upon activation, inositol-requiring enzyme 1 (IRE1) cleaves the mRNA of X-box-binding protein 1 (XBP1), producing the active cleaved form, XBP1s, thereby upregulating the expression of genes involved in ER repair [87]. However, under sustained PA stimulation, this enzyme activates inflammatory signaling pathways such as c-Jun N-terminal kinase (JNK), promoting the release of TNF-α, IL-6, and other cytokines, thereby exacerbating the progression of liver inflammation [88].

Upon activation, Activator of Transcription Factor 6 (ATF6) enters the cell nucleus and upregulates the expression of molecular chaperones such as glucose-regulated protein 78 (GRP78, also known as BiP), thereby aiding protein folding; this constitutes the compensatory repair mechanism of the endoplasmic reticulum [87]. However, if the stimulus from PA is too strong, ATF6 activation is insufficient, and GRP78’s repair capacity is limited, which will lead to the continued accumulation of misfolded proteins, exacerbating stress-induced damage [89].

ER stress has been reported to affect mitochondrial function via mitochondrial-associated endoplasmic reticulum (MAMs). Upon entering hepatocytes, PA is suggested to increase the number of MAMs, causing a massive transfer of Ca^2+^ from the ER to the mitochondria. This may trigger mitochondrial Ca^2+^ overload, disrupt the membrane potential, impair ATP synthesis, and exacerbate cellular damage [90]. This mechanism also plays a significant role in insulin resistance and diabetic complications [91].

#### 4.2.3. Dysregulation of Cellular Autophagy

Autophagy maintains cellular homeostasis by forming autophagosomes that encapsulate damaged organelles or accumulated lipids, which are then degraded after fusing with lysosomes. In a PA-induced zebrafish liver injury model, autophagy represents a cellular adaptive or pathological response to lipid accumulation and the formation of insoluble inclusions, primarily influencing downstream inflammatory and apoptotic pathways by regulating processes such as protein degradation and lipid metabolism.

Adenosine monophosphate-activated protein kinase (AMPK), acting as the cell’s energy sensor, is a key activator of autophagy; it initiates autophagy by inhibiting mTORC1. However, PA inhibits AMPK activity, leading to sustained activation of mTORC1 and resulting in impaired autophagy. Autophagy dysregulation prevents the degradation of the autophagy substrate protein p62/SQSTM1, causing it to accumulate within the cell. p62, also known as SQSTM1, is a core component of insoluble protein inclusions and plays a primary role in autophagy and protein degradation [92]. Its levels are positively correlated with hepatic lipotoxicity: the greater the accumulation of p62, the more severe the oxidative stress and inflammatory response, the more inhibited autophagy function becomes, and the more severe the liver damage. Studies have shown that p62 knockdown significantly reduces the formation of inclusions and alleviates PA-induced hepatocyte lipotoxicity [20,93], and can also effectively mitigate the adverse effects caused by defective autophagy under various pathological conditions [94,95]; conversely, p62 overexpression blocks the autophagy pathway and inhibits the clearance of abnormal substances such as excess lipids. Therefore, regulating intracellular protein levels in hepatocytes holds promise as a strategy to intervene in PA-induced hepatic lipotoxicity.

At the same time, PA may increase lysosomal membrane permeability and induce cathepsin B leakage from lysosomes into the cytoplasm. Consistent with findings in mouse macrophages [96], cytosolic cathepsin B may activate the NLRP3 inflammasome to generate mature IL-1β and IL-18, thereby triggering inflammatory infiltration in the liver. This indicates that dysregulation of autophagy is interlinked with processes such as inflammation and apoptosis.

### 4.3. Inflammatory Response and Liver Damage: From Reversible Injury to Irreversible Damage

When cellular stress exceeds the tolerance threshold of hepatocytes, it triggers the release of inflammatory mediators, culminating in inflammation and cell death. Hepatic steatosis may progress to an NAFLD-like state, characterized by both fat accumulation and infiltrative inflammation. Under sustained PA exposure and at sufficiently high doses, this process could drive a transition from reversible steatohepatitis to fibrosis. Thus, inflammation appears to represent a critical juncture at which PA-induced liver injury may shift toward more persistent damage in the zebrafish model. However, the extent to which this progression fully recapitulates human cirrhosis requires further investigation.

#### 4.3.1. Stage I: Activation of Inflammatory Signaling Pathways and Cytokine Storm

PA directly activates three core inflammatory signaling pathways—NF-κB, JNK, and p38MAPK—in hepatocytes through mechanisms involving oxidative stress and endoplasmic reticulum stress.

NF-κB is widely recognized as a central inflammatory pathway; PA activates it through ER stress and mitochondrial ROS production, thereby initiating the transcriptional upregulation of pro-inflammatory cytokines such as TNF-α and IL-1β [97]. JNK and p38MAPK, both members of the mitogen-activated protein kinase (MAPK) family, amplify the inflammatory response: JNK not only cooperates with NF-κB to promote cytokine production but also directly induces hepatocyte apoptosis [98]; p38MAPK senses oxidative damage while propagating inflammatory signals, further intensifying cellular stress. Together, these three pathways orchestrate a cytokine storm, driving the coordinated production and release of TNF-α, IL-1β, and IL-6 [40,41].

Once released, these cytokines perpetuate and amplify liver inflammation through multiple mechanisms. TNF-α directly damages hepatocyte membranes, recruits peripheral immune cells to the liver, and further activates NF-κB signaling, inducing additional IL-6 and IL-1β release. IL-1β enhances vascular permeability and sensitizes hepatocytes to TNF-α, promoting immune cell infiltration; it also has been suggested to engage in a positive feedback loop with the NLRP3 inflammasome, linking inflammation to pyroptosis. IL-6 acts as a bridge between local and systemic inflammation: locally, it promotes inflammatory cell aggregation and disrupts hepatocyte glycolipid metabolism, exacerbating lipid accumulation; systemically, it triggers a low-grade inflammatory state associated with insulin resistance. Studies in PA-fed mice have reported markedly elevated pro-inflammatory cytokine levels [99], which positively correlate with hepatic inflammatory cell infiltration [20].

#### 4.3.2. Stage II: Immune Cell Infiltration and Macrophage Polarization

The cytokine storm initiated in Stage I propagates through the bloodstream and interstitial spaces, recruiting peripheral immune cells—particularly macrophages—to accumulate in the liver [100,101]. Under the sustained injurious effects of PA, infiltrating macrophages undergo a phenotypic switch from a resting to an activated pro-inflammatory state, losing their tissue-reparative functions and instead releasing additional inflammatory cytokines and ROS, thereby exacerbating both inflammation and oxidative stress.

This macrophage-mediated amplification loop represents a hallmark feature of NASH progression [102,103,104]. Activated inflammatory macrophages promote hepatic steatosis [105], recruit inflammatory lymphocytes [106], stimulate angiogenesis [107], and critically secrete pro-fibrotic factors such as TGF-β [108]. The accumulation and inflammatory polarization of hepatic macrophages are driven by excessive lipids and free fatty acids [101], creating a self-sustaining cycle: inflammatory signaling pathways promote cytokine release, cytokines recruit and polarize macrophages, and these macrophages in turn further activate signaling pathways and release more cytokines. This positive feedback loop transforms acute inflammation into a chronic, self-perpetuating process, setting the stage for fibrosis. At this point, an NASH-like microenvironment has become established in the liver, indicating that PA-induced lipotoxicity has progressed from a reversible stage toward fibrosis and cirrhosis.

#### 4.3.3. Stage III: Hepatic Stellate Cell Activation and TGF-β/Smad-Mediated Fibrosis

A key stage in the potential progression of PA-induced liver injury is the transition from persistent inflammation to fibrosis, as reported in the available zebrafish studies—an abnormal reparative response driven primarily by the activation of hepatic stellate cells (HSCs).

HSCs are the key effector cells in liver fibrogenesis. In their quiescent state, they reside in the perisinusoidal spaces, store vitamin A in lipid droplets, and secrete only small amounts of extracellular matrix (ECM) to maintain normal liver structure. However, when subjected to persistent inflammatory stimulation—particularly by TGF-β, a potent pro-fibrotic cytokine secreted in large quantities by infiltrating macrophages and dying hepatocytes—HSCs undergo a dramatic phenotypic transition. They lose their vitamin A droplets, transform from a stellate to a spindle-shaped myofibroblast morphology, and acquire high proliferative and ECM-synthetic capacity, becoming the primary producers of scar tissue [109,110].

The TGF-β/Smad2/3 pathway is the central molecular axis governing this transition [111,112]. Upon TGF-β binding to HSC membrane receptors, downstream Smad2 and Smad3 are phosphorylated and form a complex with Smad4 [113,114]. This Smad complex translocates to the nucleus, where it specifically binds to the promoters of fibrosis-related genes, initiating transcription of α-smooth muscle actin (α-SMA)—a marker of myofibroblast activation—and of collagen synthesis genes, including COL1A1 and COL3A1 [115]. Elevated α-SMA reflects the contractile and migratory capacity acquired by activated HSCs; COL3A1 upregulation is prominent in early fibrosis, whereas COL1A1 predominates in advanced fibrosis and cirrhosis. The intensity of collagen gene expression directly correlates with the rate of ECM deposition and scar formation. Additionally, TGF-β can promote fibrosis through diverse mechanisms, including cellular senescence, metabolic reprogramming, oxidative stress, and epigenetic regulation [116].

Critically, the stage of liver fibrosis remains reversible; timely intervention can promote degradation of deposited ECM and HSC deactivation [117]. However, if the inflammatory stimulus persists, progressive scar tissue formation will extensively replace normal liver parenchyma, leading to irreversible cirrhosis and ultimately liver failure. Therefore, Stage III represents the critical therapeutic window for halting NAFLD progression—a transition that is not merely a passive consequence of inflammation, but an active, HSC-driven process that can be therapeutically targeted.

### 4.4. Specific Advantages of the Adult Zebrafish Model for PA-Induced Hepatotoxicity Studies

The adult zebrafish model holds irreplaceable value in studying PA-induced chronic liver injury, particularly in simulating the long-term progression of human NAFLD, multi-tissue interactions, transgenerational effects, and translational research—advantages that are difficult to fully replicate with cell lines or mammalian models. Feeding adult zebrafish with PA-supplemented diets can effectively recapitulate the key features of chronic liver injury within a relatively short time. Compared with mouse models, which are costly and time-consuming, the zebrafish model more faithfully mimics the progressive pathogenesis of human NAFLD. It also supports high-density housing, flexible dosing, and multi-time-point experimental designs, facilitating efficient time-effect analysis.

Adult zebrafish possess a complete metabolic network, in which liver injury is closely associated with multiple systems, including the gut microbiota and adipose tissue [118]. Notably, adult zebrafish harbor a mature gut microbiota structurally similar to that of mammals, enabling direct verification of the causal role of specific microbial communities in PA-induced hepatotoxicity via the gut-liver axis. Indeed, studies have demonstrated that PA exposure significantly alters gut microbiota composition, disrupts the intestinal barrier, promotes endotoxin translocation, and consequently exacerbates hepatic inflammation and steatosis [42].

The short generation time (approximately 3 months) and high fecundity of adult zebrafish make them uniquely suited to test transgenerational transmission of PA-induced NAFLD susceptibility—a task that would take years and massive resources in mice, yet can be completed within one year in zebrafish. Studies show that 4-tert-Butylphenol-induced parental liver damage remodels offspring hepatic DNA methylation, conveying metabolic memory across generations [119].

Adult zebrafish exhibit hepatic pathology and conserved molecular pathways that closely resemble those in human NAFLD/NASH. Through simple immersion or dietary dosing, they enable medium-throughput in vivo efficacy testing with concurrent assessment of serum biomarkers, histopathology, and inflammatory markers, making them an efficient bridge between cell-based screening and mammalian models—addressing the limitations of cell cultures in organ crosstalk while offering practical advantages over mice for early pharmacodynamic and toxicological studies.

Using the adult zebrafish model, researchers have reported several original findings. For example, PA dietary exposure induces hepatic steatosis, hyperlipidemia, and hyperglycemia in adult zebrafish [17]; the dynamic process of PA-induced liver fibrosis was successfully observed in living adult fish, identifying p62 accumulation as a key hallmark of lipotoxic injury [20]; and the model first revealed the gut microbiota-mediated mechanism of PA hepatotoxicity, enabling screening of natural compounds (e.g., diacylglycerol, curcumin, glycyrrhizic acid) that effectively ameliorate lipid dysregulation. Collectively, these findings demonstrate that the adult zebrafish is not only a reliable platform for mechanistic studies but also a valuable source for discovering novel targets and intervention strategies.

**Figure 3:**
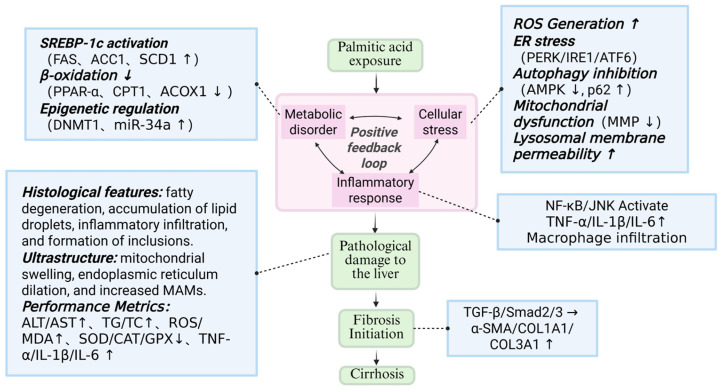
Molecular mechanisms underlying PA-induced liver injury in zebrafish. Footnotes: PA disrupts hepatic lipid homeostasis via three interconnected pathological layers—metabolic disorder, cellular stress, and inflammatory/fibrotic response—forming a positive feedback loop. Solid arrows: direct regulation; dashed arrows: indirect/multi-step regulation; upward arrows (↑): increased expression or activation; downward arrows (↓): decreased expression or inhibition. For detailed evidence, see Table 2. Data compiled from mechanistic studies on PA-induced hepatic lipotoxicity [48,49,50,51,60,61,62,63,66]. Created in BioRender. Li, W. (2026). https://biorender.com/cc4lej8 (accessed on 21 April 2026).

The molecular mechanism underlying PA-induced liver injury in zebrafish is shown in Figure 3.

The molecules and signaling pathways involved in PA-induced liver injury in zebrafish are shown in Table 1.

The experimental model origins of each key mechanism are summarized in Table 2.

## 5. Research Progress on Intervention Strategies for PA- and Other Saturated Fatty Acid-Induced Hepatotoxicity in Zebrafish

Current intervention strategies against palmitic acid (PA)-evoked hepatic lipotoxicity fall into three core categories: correcting metabolic disorders, alleviating cellular stress, and suppressing inflammatory cascades. Available intervention agents cover small-molecule inhibitors, natural bioactive compounds, epigenetic modulators, and nutritional additives. To enable stratified presentation of evidence, this section rigorously categorizes research findings: interventions validated via vivo assays in adult zebrafish are presented first as Tier 1 robust evidence, while data derived from larval zebrafish, mammalian cell lines, and rodent models serve only as indirect supportive evidence.

### 5.1. Interventions to Address PA-Induced Metabolic Disorders in Zebrafish Livers

#### 5.1.1. Inhibiting Lipid Synthesis Pathways

AMPK acts as a master energy sensor with great therapeutic potential for metabolic and inflammatory disorders [120]. Multiple natural compounds alleviate hepatic lipid accumulation in adult zebrafish via the AMPK–SREBP-1c axis, targeting distinct pathways. β-sitosterol blocks the PPARγ/RXR lipogenic cascade [121]. As AMPK agonists, leonurine hydrochloride [39] and trans-cinnamic acid [122] repress de novo lipogenesis by reducing the transcriptional activity of SREBP-1c. Peanut-derived diacylglycerol bidirectionally regulates hepatic lipid anabolism and catabolism to restore systemic lipid homeostasis in adult zebrafish [123].

Consistent observations of this pathway have been reported in alternative experimental models. Baicalin has been reported to inhibit the mTORC1 pathway and downregulate SREBP-1c expression in mammalian cell lines [124]. Geniposide reduces SREBP-1c abundance in larval zebrafish and AML12 hepatocytes [125]. A picroside-rich fraction suppresses the transcription of lipogenic genes *lipin1* and *cidec* [126]. The herbal formula Si-Ni-San attenuates hepatic lipid deposition by inhibiting *Fasn* via AMPK signaling in mice [127]. Nevertheless, none of these agents have been tested for efficacy or safety in adult zebrafish models of PA-induced liver injury; they merely provide preliminary mechanistic hypotheses rather than direct application references.

Current zebrafish studies only qualitatively describe lipid-lowering phenotypes without standardized dose–response profiles. Most compounds lack specific binding affinity for SREBP-1c and may disrupt basal lipid synthesis, which is essential for hepatic physiological function. The long-term hepatic toxic risks arising from sustained lipogenic inhibition remain uncharacterized. Further experiments with gradient dosing and off-target profiling are required for comprehensive validation.

#### 5.1.2. Promote Fatty Acid Oxidation

Activation of the PPAR-α signaling axis is the core therapeutic target for reversing palmitic acid (PA)-mediated suppression of fatty acid catabolism, accelerating lipid clearance in hepatocytes, and re-establishing metabolic homeostasis. In adult zebrafish, senkyunolide I simultaneously upregulates PPARα and downregulates fatty acid transporter CD36, exerting dual effects to reduce hepatocellular fatty acid uptake and expedite mitochondrial β-oxidation [128]. Two rate-limiting enzymes govern this catabolic process: CPT1A mediates the transport of long-chain fatty acids into mitochondria, while ACOX1 is responsible for peroxisomal breakdown of very long-chain fatty acids. The active bitter melon extract TCD restores impaired mitochondrial fatty acid transport in adult zebrafish by reversing high-fat diet-induced CPT1A repression [129].

Studies using alternative models have yielded complementary results. *Polygonum multiflorum* and its constituent emodin upregulate AMPKα and PPAR-α to elevate CPT1A and ACOX1 expression in larval zebrafish [130]. However, emodin has been shown to be hepatotoxic in mammals and thus requires cautious use. Lingonberry extracts [131], soybean insoluble dietary fiber [132], and aurantio-obtusin [133] facilitate fatty acid oxidation in mammalian cell and animal models.

From a critical perspective, existing pro-oxidative interventions assess only short-term hepatic lipid markers and lack longitudinal data on disturbances in adaptive energy metabolism following sustained treatment. PPAR transcription factors display substantial interspecies divergence, meaning agonist effects observed in mammals cannot be directly extrapolated to teleost fish. Nevertheless, research targeting fatty acid oxidation lays a solid theoretical foundation for the application of plant-derived lipids and clinical treatment of human NAFLD/NASH.

#### 5.1.3. Epigenetic Intervention Strategies

The core objective of epigenetic intervention is to reverse persistent liver damage driven by palmitic acid (PA)-mediated “metabolic memory”. It restores the expression of silenced fatty acid oxidation genes via regulating DNA methylation and non-coding RNAs. Research on adult zebrafish delivers meaningful evidence: parental diets rich in arachidonic acid remodel hepatic DNA methylomic landscapes of progeny, confirming that lipotoxicity can be inherited across generations through epigenetic modifications [134]. Two effective epigenetic regulators validated in adult zebrafish are listed below: the *Slc25a1*-specific inhibitor CTPI-2 remodels histone and non-histone acetylation, modulates sirtuin expression, and reverses the silencing of lipid metabolic genes [135]; folate rescues m6A RNA hypomethylation induced by combined triclosan and PA exposure, and significantly alleviates hepatic metabolic disorders in adult zebrafish [136].

Findings from other experimental models reveal the therapeutic potential of non-coding RNA-based strategies. Liver-specific knockdown of miR-7a [137] or miR-27b [138] triggers NAFLD-like pathological phenotypes. In parallel, 5-azacytidine has been shown to restore PPAR pathway activity through promoter demethylation in cell lines [139], and mechanistic studies using methyltransferase inhibitors and gene knockdown have identified DNA 5mC and RNA m6A modifications as sequential regulators of hepatic stellate cell activation during liver fibrosis, highlighting these epigenetic marks as potential therapeutic targets [140]. Moreover, epigenetic therapeutics have advanced rapidly for other illnesses. Natural products such as green tea polyphenols [141], curcuminoids [142], and terpenoids [143] exert epigenetic regulatory effects in tumor models, and epigenetic interventions have entered clinical exploratory research for cancers [144], neurological [145], and immune disorders, which proves that epigenetic modulation is a vibrant and promising research avenue.

However, epigenetic modulators act on broad targets and carry high off-target risks. Most existing studies focus solely on individual epigenetic modifications, without delineating crosstalk among multiple regulatory pathways. Meanwhile, substantial differences exist between the epigenetic regulatory networks of zebrafish and humans. Therefore, relevant results remain confined to preclinical basic research, with considerable translational gaps in the clinical treatment of human NAFLD.

### 5.2. Interventions for Cellular Stress

#### 5.2.1. Antioxidant Therapy

Classical hepatoprotective antioxidants cover vitamin E, silymarin, and N-acetylcysteine. Nrf2 agonists (sulforaphane, resveratrol), selenium supplements and mitochondria-targeted coenzyme Q10 also exhibit antioxidant activity. Multiple natural bioactive compounds have been proven to regulate oxidative stress and ameliorate lipid metabolism in adult zebrafish: leonurine hydrochloride [39], peanut-derived diacylglycerol [123], lychee peel powder [146] and carnauba wax 4-methoxycinnamic acid diester [147] lower hepatic reactive oxygen species (ROS) and malondialdehyde (MDA) contents while elevating the activities of antioxidant enzymes SOD and GPx; gallic acid modulates purine metabolic pathways to relieve oxidative tissue damage [148].

Studies using alternative models demonstrate that α-tocopherol (vitamin E) exerts antioxidant effects in juvenile aquatic species [149], whereas ellagic acid merely protects zebrafish embryos from oxidative DNA lesions [150]. Most relevant experiments use short-term acute exposure schemes and lack chronic safety data for long-term dietary supplementation. Moreover, all antioxidants have narrow therapeutic windows between effective and toxic concentrations, demanding further in-depth research. Nonetheless, well-characterized antioxidants have been widely adopted in human clinical medicine, livestock breeding and veterinary medicine. As typical examples, silymarin and its monomer silybin extracted from milk thistle are renowned hepatoprotectors that have been put into mass production and daily application.

#### 5.2.2. Mitigation of Endoplasmic Reticulum (ER) Stress

Direct approaches to relieve ER stress focus on supplementing molecular chaperones such as taurocholic acid, 4-phenylbutyric acid, taurine. In vivo experiments on adult zebrafish, curcumin [151], resveratrol [152], and silymarin [153] suppress overactivation of the PERK/IRE1/ATF6 ER stress cascade, thereby alleviating misfolded protein accumulation and hepatocyte apoptosis.

Studies based on alternative models show that mycophenolate mofetil and ursodeoxycholic acid block palmitic acid (PA)-induced apoptosis in primary mouse hepatocytes [154]; 4-phenylbutyric acid ameliorates hepatic ER damage in high-fat diet-fed rats and mice [155,156]. These results collectively demonstrate that natural products exert hepatoprotective effects through multiple synergistic molecular mechanisms rather than a single independent pathway. Further academic research is needed to differentiate core therapeutic targets from auxiliary regulatory factors in this signaling network.

#### 5.2.3. Restoring Autophagy Balance and p62 Regulation

Research on adult zebrafish confirms p62 as a key target of PA-triggered hepatic lipotoxicity: genetic knockdown of p62 markedly eliminates PA-derived insoluble protein inclusions and simultaneously alleviates hepatic oxidative stress and inflammatory injury [20]. Isarubrolone C induces hepatic lipophagy through an AMPK-dependent pathway in zebrafish [157].

Data from other experimental models indicate that astragalin initiates ferritinophagy to protect livers in larval zebrafish [158]. The Qigui Jiangzhi herbal formula amplifies autophagy via the AMPK/SIRT1/TFEB axis in mammalian models [159]. Intact fusion and degradation of autophagosomes and lysosomes are prerequisites for functional autophagy [160]. Quercetin [161] and resveratrol [162] function as lysosomal protectants to recover lysosomal activity in cell lines. Functional teas from *Penthorum chinense* Pursh downregulate p62 and upregulate autophagic signaling in mammalian cells and animal models [163].

Comprehensive analysis reveals that although p62 knockdown clears insoluble protein aggregates, p62 participates in multiple signaling pathways unrelated to autophagy, leading to insufficient target specificity for monotherapy. Moreover, autophagy exerts bidirectional regulatory effects, and its overactivation may trigger hepatocyte autophagic death. Accordingly, the safe therapeutic concentration range requires precise characterization in follow-up studies.

### 5.3. Interventions Targeting Inflammatory Responses

In studies using adult zebrafish, glyceryl monolaurate suppresses the transcription of pro-inflammatory mediators IL-1β, IL-8, NF-κB, p65, and TNF-α while elevating anti-inflammatory TGF-β1 [164]. Peanut-derived diacylglycerol downregulates hepatic Il-1β and mitigates inflammatory immune cell infiltration [123]. The polyphenol diphlorethohydroxycarmalol broadly inhibits the expression of IL-1β, IL-6, TNF-α, NF-κB, and COX-2 in zebrafish livers [165]. Consistent with the molecular mechanisms elaborated above, anti-inflammatory approaches validated in adult zebrafish cover three major strategies: signaling pathway blockade, cytokine neutralization, and macrophage phenotype modulation. Most bioactive agents achieve maximal anti-inflammatory efficacy through synergistic multi-target regulation, which is of practical value for aquaculture production.

Studies on alternative experimental models report that picroside-rich fractions [126], sesamin [166], and folic acid [167] block the NF-κB and NLRP3 inflammatory cascades respectively. Apigenin facilitates the polarization of anti-inflammatory M2 macrophages in mice [168]. Nevertheless, therapeutic strategies based on macrophage polarization have not been validated in adult zebrafish, and systematic research on M1/M2 biomarkers and physiological functions in teleost fish remains insufficient.

### 5.4. Critical Evaluation of Intervention Strategies

Although the intervention strategies discussed above have demonstrated certain potential in adult zebrafish models, several noteworthy issues remain regarding their practical application and translational development. The following provides a brief evaluation from four aspects: effective concentration variability, safety risks, practical applicability, and translational potential.

First, the effective concentrations of bioactive compounds validated in adult zebrafish vary considerably. Leonurine hydrochloride and trans-cinnamic acid exert hepatoprotective effects at micromolar concentrations [39,122], whereas phytosterols such as β-sitosterol require much higher dietary supplementation levels to achieve efficacy [121]. This heterogeneity, combined with the lack of standardized administration routes, PA modeling durations, and detection endpoints across different studies, prevents direct comparison of protective efficacy among various interventions. Therefore, the establishment of standardized protocols for PA-induced liver injury modeling and pharmacological evaluation is urgently needed to facilitate the integration of research findings.

Second, many natural extracts with hepatoprotective potential carry dose-dependent safety risks. For example, emodin has been confirmed to cause liver damage in mammals [130], and high-concentration quercetin shifts from antioxidant to pro-oxidant activity [161]. Consequently, comprehensive toxicological evaluation must be performed to define the safe dosage range before any candidate additive is considered for aquaculture or preclinical research.

From a practical application perspective, certain lipid-lowering agents—such as diacylglycerol and glycerol monolaurate—offer advantages in raw material cost and feed processing stability, making them promising candidates for industrial aquafeed production. In contrast, polyphenols and herbal extracts face challenges for large-scale promotion in the short term due to high extraction costs and susceptibility to oxidative inactivation during storage and processing.

Regarding translational potential, the zebrafish model serves as a valuable early-stage platform for generating mechanistic hypotheses and screening lead compounds. Although it cannot fully reproduce the systemic metabolic derangements of human NAFLD, and interspecies pharmacokinetic differences preclude direct dose extrapolation from fish to humans, its medium-throughput in vivo efficacy testing capability provides a critical bridge between cell-based assays and mammalian studies. Positive intervention results obtained in zebrafish should be viewed as promising translational starting points that require further validation in rodent models, rather than as definitive evidence for human application; nevertheless, this does not diminish the model’s important role in accelerating the discovery and preliminary evaluation of potential therapeutic agents.

The model origins of each intervention are summarized in Table 3.

## 6. Conclusions and Future Directions

### 6.1. Limitations

#### 6.1.1. Methodological Limitations of the Review Process

The literature screening was performed by a single reviewer (W.L.) in the initial stage, followed by cross-verification by three collaborators (K.J., N.A., and S.P.). However, the standard procedure of independent dual screening by two reviewers was not implemented. Formal blinding of reviewers to author identities and institutional affiliations was not applied, and inter-reviewer agreement was not quantitatively assessed (e.g., via Kappa statistics). In addition, this review did not employ standardized risk-of-bias assessment tools (e.g., SYRCLE’s Risk of Bias tool for animal studies) or the GRADE evidence grading system.

The primary rationale for these methodological choices is that this review is positioned as a comprehensive qualitative synthesis of the molecular mechanisms and signaling pathways underlying PA-induced hepatic injury, rather than a quantitative meta-analysis of effect sizes for specific interventions. Readers should be aware of these methodological characteristics when interpreting the evidence synthesized in this review. Future systematic reviews on specific interventions should consider incorporating standardized quality appraisal tools such as SYRCLE’s Risk of Bias tool.

#### 6.1.2. Heterogeneity in PA Exposure Protocols Across Studies

Substantial variation exists across studies in PA concentration, route of administration (dietary supplementation vs. waterborne exposure), exposure duration, and dietary background (e.g., high-fat diet composition). Such heterogeneity complicates the direct comparison and integration of pathological outcomes across different studies. The establishment of relatively standardized exposure protocols would facilitate cross-study comparability and data synthesis.

#### 6.1.3. Evidence Gaps: Mechanisms Requiring Direct Validation in Adult Zebrafish

It should be noted that several molecular mechanisms discussed in this review—such as NADPH oxidase-mediated ROS production, mitochondrial-associated ER membranes (MAMs)-mediated Ca^2+^ transfer, and NLRP3 inflammasome activation—are currently supported primarily by mammalian or cell-based studies, and direct experimental evidence from adult zebrafish is yet to be established. These mechanisms are included in this review to provide testable hypotheses and guidance for future investigations in adult zebrafish, rather than as established conclusions in this model.

### 6.2. Conclusions

This review systematically examines the pathological features of PA-induced hepatic lipotoxicity using an adult zebrafish model; it reveals a comprehensive molecular pathway ranging from metabolic dysregulation and cellular stress to inflammation and fibrosis; building on this foundation, this review summarizes therapeutic strategies across multiple avenues, including inhibition of lipid synthesis, promotion of fatty acid oxidation, epigenetic regulation, antioxidant measures, alleviation of cellular stress, and anti-inflammatory approaches; in the future, these strategies can be combined with technologies such as gene editing (CRISPR/Cas9), single-cell sequencing, and spatial transcriptomics to lay the theoretical groundwork for the development of novel interventions and targeted drugs.

### 6.3. Future Directions

Despite the progress summarized above, several key scientific issues remain to be addressed. First, the interactive crosstalk among metabolic disorders, cellular stress, inflammation, and epigenetic regulation needs to be systematically elucidated, particularly the temporal dynamics and regulatory networks connecting these pathological layers. Second, the integration of multi-omics technologies—including transcriptomics, metabolomics, proteomics, and single-cell sequencing—holds promise for identifying novel biomarkers and therapeutic targets at higher resolution. Third, the long-term persistence and reversibility of PA-induced epigenetic modifications and metabolic memory warrant further investigation through longitudinal and transgenerational studies to determine the optimal timing for intervention. Addressing these questions will not only advance our understanding of the fundamental mechanisms of lipotoxic liver injury but also facilitate the development of practical strategies for both aquaculture and clinical management of NAFLD.

## Figures and Tables

**Table 1:** A Brief Overview of Key Molecules/Signaling Pathways Involved in PA-Induced Liver Injury in Zebrafish and Their Roles.

Category	Key Molecules /Pathways	Regulation in PA Model	Brief Description	Representative Evidence
Metabolic disorder	SREBP-1c /FAS/ACC1/SCD1	↑	Enhanced lipid synthesis	[48,49,50,51]
	PPAR-α /CPT1/ACOX1	↓	β-oxidation inhibition	[60,61,62,63,66]
	DNMT1/miR-34a	↑	Epigenetic silencing of oxidative genes	[73,74,75,76,77,78]
Cellular stress	ROS/SOD /CAT/GPX	↑/↓	Oxidative stress	[37,38,79,80,82]
	PERK/IRE1/ATF6	↑	ER stress/UPR activation	[84,85,86,87,88,89]
	AMPK/p62 /Lysosomes	↑/↓	Autophagy dysregulation	[20,92,93,94,95,96]
Inflammatory & fibrotic	NF-κB/JNK/p38MAPK	↑	Inflammatory signals are activated	[40,41,97,98]
	TNF-α/IL-1β/IL-6	↑	Inflammatory cytokines are released	[40,41,99]
	Macrophage infiltration	↑	Inflammation is amplified	[102,103,104]
	TGF-β/Smad2/3/α-SMA	↑	Activation of hepatic stellate cells	[111,112,113,114,115,116]

↑, upregulation or activation; ↓, downregulation or inhibition. “Regulation in PA Model” indicates the reported direction of change in PA-exposed zebrafish or mammalian models. For full abbreviations, see the Abbreviations section.

**Table 2:** Evidence Source Summary for Key Mechanisms.

Mechanism Category	Validated in Adult Zebrafish	Supported by Other Models (Not Yet Validated in Adult Zebrafish)
Lipid synthesis (SREBP-1c/FAS/ACC1)	[17,20]	[48,49,50,51]
β-oxidation inhibition (CPT1/PPAR-α)	[20]	[60,61,62,63,66]
Epigenetic regulation (DNMT1/miR-34a/lncRNA)	[73,78]	[74,75,76]
Oxidative stress & ROS	[17,34]	[79,80,81]
ER stress & UPR	[20,40]	[84,85,87,88]
Autophagy & p62 accumulation	[20]	[92,94,95]
Inflammatory signaling (NF-κB/JNK)	[40,41]	[97,98,99]
Macrophage infiltration & fibrosis	[22,109,110]	[100,101,102,103,104,109,110,111,112,113,114,115,116]

This table summarises experimental model origins for mechanisms discussed in this review. “Validated in Adult Zebrafish” indicates support by at least one original study using adult zebrafish; “Other Models” includes larvae/embryos, mammalian models, and cell lines, cited for mechanistic illustration only.

**Table 3:** Evidence Source Summary for Key Interventions.

Intervention Category	Validated in Adult Zebrafish	Supported by Other Models (Larvae/Mammalian/Cell Lines)
Lipid synthesis inhibitors (AMPK activators, SREBP-1c inhibitors)	[39,120,121,122,123]	[124,125,126,127]
Fatty acid oxidation promoters (PPAR-α agonists)	[128,129]	[130,131,132,133]
Epigenetic modulators (DNMT inhibitors, miRNA mimics, m6A modulators)	[134,135,136]	[137,138,139,140,141,142,143,144,145]
Antioxidants	[39,146,147,148]	[149,150]
ER stress relievers	[151,152,153]	[154,155,156]
Autophagy regulators (p62 modulators, autophagy activators)	[20,157]	[158,159,160,161,162,163]
Anti-inflammatory agents	[123,164,165]	[126,166,167,168]

This table summarises the experimental model origins for the intervention strategies discussed in this review. “Validated in Adult Zebrafish” indicates that the intervention has been tested in vivo in adult zebrafish and shown efficacy against PA-induced hepatic lipotoxicity; “Other Models” includes larval zebrafish, mammalian models, and cell lines, cited as complementary or supporting evidence only.

## Data Availability

Schematic diagrams (Graphical Abstract, Figure 2 and Figure 3) were created using BioRender.com under a licensing agreement. All molecular and pathology-related data were compiled from original research papers cited in the reference list, and the relevant references can be found in the manuscript’s reference section. The findings of this study are published under the Creative Commons Attribution 4.0 International Open Access License (CC BY 4.0).

## Abbreviations

The following abbreviations are used in this manuscript:

ACC1	Acetyl-CoA Carboxylase 1
ACOX1	Acyl-CoA Oxidase 1
ALP	Alkaline Phosphatase
ALT	Alanine Transaminase
AMPK	Adenosine Monophosphate-Activated Protein Kinase
AST	Aspartate Transaminase
ATF4	Activating Transcription Factor 4
ATF6	Activating Transcription Factor 6
BiP	Binding Immunoglobulin Protein
CAT	Catalase
COL1A1	Collagen Type I Alpha 1
COL3A1	Collagen Type III Alpha 1
CPT1	Carnitine Palmitoyltransferase 1
CPT1A	Carnitine Palmitoyltransferase 1A
DNMT1	DNA Methyltransferase 1
ECM	Extracellular Matrix
ER	Endoplasmic Reticulum
FAS	Fatty Acid Synthase
GPX	Glutathione Peroxidase
GRP78	Glucose-Regulated Protein 78
GGT	Gamma-Glutamyl Transferase
HSCs	Hepatic Stellate Cells
IL-1β	Interleukin-1β
IL-6	Interleukin-6
IL-10	Interleukin-10
IRE1	Inositol-Requiring Enzyme 1
JNK	c-Jun N-terminal Kinase
MAMs	Mitochondria-Associated ER Membranes
MDA	Malondialdehyde
MMP	Mitochondrial Membrane Potential
mTORC1	Mammalian Target of Rapamycin Complex 1
NAFLD	Non-Alcoholic Fatty Liver Disease
NASH	Non-Alcoholic Steatohepatitis
NLRP3	NOD-Like Receptor Family Pyrin Domain Containing 3
NOX	NADPH Oxidase
PA	Palmitic Acid
PERK	Protein Kinase R-like ER Kinase
PGC-1α	Peroxisome Proliferator-Activated Receptor Gamma Coactivator 1α
PPAR-α	Peroxisome Proliferator-Activated Receptor α
PPAR-γ	Peroxisome Proliferator-Activated Receptor γ
ROS	Reactive Oxygen Species
SCD1	Stearoyl-CoA Desaturase 1
SFA	Saturated Fatty Acid
SIRT1	Sirtuin 1
SIRT3	Sirtuin 3
Smad2	Mothers Against Decapentaplegic Homolog 2
Smad3	Mothers Against Decapentaplegic Homolog 3
SOD	Superoxide Dismutase
SREBP-1c	Sterol Regulatory Element-Binding Protein-1c
TC	Total Cholesterol
TG	Triglyceride
TGF-β	Transforming Growth Factor-β
TNF-α	Tumor Necrosis Factor-α
UPR	Unfolded Protein Response
VLDL	Very-Low-Density Lipoprotein
XBP1	X-Box Binding Protein 1
XDH	Xanthine Dehydrogenase
XO	Xanthine Oxidase
α-SMA	Alpha-Smooth Muscle Actin
